# Harm or protection? The adaptive function of tick toxins

**DOI:** 10.1111/eva.13123

**Published:** 2020-09-29

**Authors:** Péter Apari, Gábor Földvári

**Affiliations:** ^1^ Institute of Evolution Centre for Ecological Research Budapest Hungary

**Keywords:** adaptive function, antimicrobial effect, necroptosis, protease inhibitors, soft and hard ticks, tachycardia, tick paralysis, tick toxins

## Abstract

The existence of tick toxins is an old enigma that has intrigued scientists for a long time. The adaptive value of using deadly toxins for predatory animals is obvious: they try to kill the prey in the most effective way or protect themselves from their natural enemies. Ticks, however, are blood‐sucking parasites, and it seems paradoxical that they have toxins similar to spiders, scorpions and snakes. Based on published data, here we examine the potential adaptive function of different types of toxins produced by soft and hard ticks. We hypothesize that there are diverse evolutionary roles behind (a) to attack and reduce the tick‐transmitted pathogens inside the vertebrate host systemically to protect the tick, (b) to paralyse the host to stop grooming, (c) to speed up host heartbeat to improve blood supply and (d) to inhibit the process of necroptosis to prevent the rejection of hard ticks. We will provide published evidence that supports the above‐mentioned hypotheses, and we will give an outlook how these new scientific results might be applied in modern pharmacology and medicine.

## 1 INTRODUCTION

**FIGURE 1:**
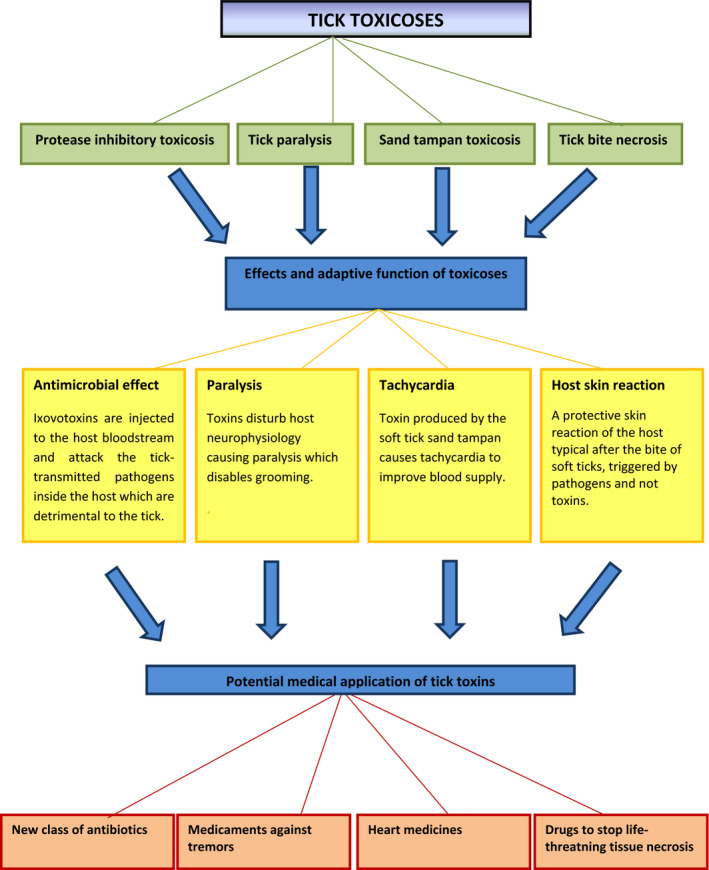
Overview of the main types of tick toxicoses and their mechanism, adaptive function and possible medical application

Ticks (Acari: Ixodida) are obligatory blood‐sucking arthropods attaching to their vertebrate hosts for short periods (soft ticks, Argasidae) or several days (hard ticks, Ixodidae). Hard ticks are well known for transmitting a large variety of pathogenic microorganisms to humans and animals causing diseases, for example Lyme borreliosis, anaplasmosis, tularemia and babesiosis (Sonenshine & Roe, 2013). In addition to blood loss and pathogen transmission, they were shown to produce toxins that are very similar to the toxins of two close relatives of ticks namely spiders and scorpions (Cabezas‐Cruz & Valdés, 2014; Cordeiro, Amorim, Anjolette, & Arantes, 2015). Tick toxins are noninfectious components that are injected into the host during feeding causing pathological changes to the host (Bowman & Nuttall, 2004). Tick toxicoses can manifest in various forms, the best known is tick paralysis and there are three other different types (Figure 1). The second one is due to a systemic toxin which has serine protease inhibitor activity and disturbs the normal function of host serine proteases, thus possibly causing adverse health consequences. The responsible toxins differ functionally, and there is evidence that the source of the second type is the egg of the ixodid ticks; therefore, it is called ixovotoxin (Mans, Gothe, & Neitz, 2004). There have been two further tick toxicoses described: one causing tachycardia and another causing tick bite necrosis in the host (Kallini & Khachemoune, 2017; Mans, Steinmann, Venter, Louw, & Neitz, 2002).

Understanding the existence of tick toxins in the light of evolutionary theory is not trivial. The fact that predatory animals produce deadly toxins is evident because of the importance to kill the prey in the most effective way and to protect the organism from their natural enemies. But being blood‐sucking parasites, ticks depend on the well‐being of their vertebrate host organism. In this article, we use a new approach applying evolutionary theory and propose new hypotheses for the possible adaptive value of tick toxins. Furthermore, we propose possible medical and pharmaceutical applications of these peculiar molecules.

## 2 PROTEASE INHIBITOR‐BASED TICK TOXICOSES

Protease inhibitor toxins have a systemic effect in contrast to the local pathogenic effect of paralysis toxins that primarily affect the nervous system (Sonenshine & Roe, 2013). Ixovotoxins are exclusively protease inhibitors, and these molecules are very effective against various pathogenic organisms (Blisnick, Foulon, & Bonnet, 2017). But what can be the adaptive value of a toxin that disturbs the normal function of protease enzymes? According to the most accepted hypothesis so far, the possible role of protease inhibitor toxins is the modulation by the host immune response, thus inhibition of tick rejection (Blisnick et al., 2017). However, ticks not known to cause toxicosis are usually not detached by host immune reaction either.

Previously, it was assumed that the function of ixovotoxins is to protect the eggs from the invasion of pathogenic microbes and have developmental and physiological roles (Bowman & Nuttall, 2004). The name ixovotoxin is particularly apt since egg toxins seem to be limited to hard or ixodid ticks. Tick egg extracts from 17 ixodid species tested were toxic, while extracts from 5 argasid species were not (Bowman & Nuttall, 2004).

Ixovotoxins are produced in exceptionally large amounts (1,000 times higher concentration per egg; Mans et al., 2004). Given the fact that ixodid ticks produce several thousand eggs (Bowman & Nuttall, 2004), this extreme high toxin concentration is energetically too costly for the protection of the eggs and the tick only. We argue that the large dose of toxin needs to provide additional benefits. A resolution of this paradox could be that the large amount of toxic molecules might be used as a preventive strike against pathogens within the considerably larger host. This enables ticks to fight against pathogens before they enter the tick tissues during feeding. In proximate terms, the usage of the eggs for the toxin production can be explained by the existence of the large number of these toxin‐producing units that are not available in other types of tick organs. It has to be noted that the antimicrobial effect of ixovotoxins needs to be selective, since ticks have endosymbiotic microbes that are essential for their survival (Buysse, Plantard, McCoy, Duron, & Menard, 2019). The assumed selectivity of tick toxins against different microbes needs to be further examined. Furthermore, experimental studies could test our prediction associated with the antimicrobial hypothesis by showing the transport of ixovotoxins from the developing eggs of the feeding female tick into its saliva.

We hypothesize that the main function of protease inhibitor tick toxins is to systemically attack and kill the tick‐transmitted pathogens inside the vertebrate host to protect ticks from the adverse effects of these microbes. Thus, tick toxicosis triggered in the host can be considered as merely an unintended by‐product of the protease inhibitor molecules of the feeding tick. But why do ticks need to protect themselves from pathogens that they are known to transmit? According to the traditional viewpoint, ticks and tick‐transmitted pathogens are in a low virulent relationship and pathogens do not harm their vector organism seriously (Ewald, 1994). However, several experimental data exist about the detrimental, sometimes lethal effect of tick‐borne pathogens onto ticks themselves. Various species of *Rickettsia, Borrelia* and *Babesia* were shown to cause impaired embryonic development, reduced fitness and fertility of female ticks or decreased rate of progeny survival (Burgdorfer, Hayes, & Corwin, 1989; Niebylski, Peacock, & Schwan, 1999; Rachinsky, Guerrero, & Scoles, 2007; Socolovschi, Mediannikov, Raoult, & Parola, 2009). In addition, the existence of a complex tick immune system (Hajdusek et al., 2013) and increasing data about the adverse effects of these microorganisms onto ticks (Blisnick et al., 2017) forces us to consider many of tick‐borne microbes as possible pathogens and not ignorable from the point of view of ticks. If we add that many tick‐borne pathogens are able to spread both vertically and horizontally, it becomes evident that transovarially transmitted bacteria can easily afford to be virulent inside the tick (Harris et al., 2017).

According to Mans et al. (2004), in the case of the hard tick species *Hyalomma truncatum,* the strains causing symptoms of sweating sickness (a tick toxicosis) were infected with pathogenic *Rickettsia* spp. On the other hand, strains that do not induce sweating sickness were negative for the pathogen. This indicates that the sweating sickness toxins in this case are directly connected to the presence of tick‐pathogenic bacteria and not to vertebrate immune system modulation. After immunizing the experimental animals with tick extract, the symptoms disappeared but the ticks took blood meal without complications on the immunized animals. Was the tick toxin immunosuppressive, the immunization would lead to rejection of the tick by the host immune system. The outcome of this experiment clearly indicates that the toxins’ primary effect is not immunomodulation.

If the primary function of these toxins was to modulate host immune response, we would expect that they are produced continuously and not occasionally. However, in the case of *Rhipicephalus microplus,* only the larvae produce toxins (Mans et al., 2004), the nymphs and the adult ticks do not (this tick has only one host during its lifetime). This indicates that the molecules’ primary function is probably not connected to immune suppression but something else. Moreover, the fact that only the larvae produce the toxin indicates an optimization of tick life history not to lose resources as an adult tick in the form of expensive toxin production.

Kunitz domains are the active domains of proteins that inhibit the function of protein‐degrading enzymes or, more specifically, domains of Kunitz‐type are protease inhibitors. (Corral‐Rodríguez, Macedo‐Ribeiro, Barbosa Pereira, & Fuentes‐Prior, 2009). Despite structural similarities, Kunitz‐type serine protease inhibitors are reported to exhibit a wide variety of biological functions, such as inhibition of one or more serine proteases, blocking of ion channels, interference with blood coagulation, inflammation and fibrinolysis (Mukherjee, Mackessy, & Dutta, 2014). Interestingly, in the tick *Ixodes scapularis* the group I Kunitz protease inhibitor evolved faster than the derived group II and III proteins, the primary function of which is to modulate ion channels and lost their protease inhibitor activity (Dai, Zhang, & Huang, 2012). This can be interpreted by evolutionary arms race between group I Kunitz protease inhibitor proteins and the large variety of pathogens present in the tick.

Ticks share the antimicrobial compounds of spiders’ and scorpions’ toxins, while other components of toxic material are absent in ticks and are specific in the other animal groups (Cordeiro et al., 2015). The presence of the toxin in both predatory and parasitic chelicerates and the importance of its antimicrobial function in the previously mentioned predators as a prey disinfectant confirm our hypothesis that antimicrobial function is one of the major roles of tick ixovotoxins.

The toxic effect of protease inhibitors is probably a by‐product because hard ticks take blood meal only once; therefore, there is little selection against the collateral damage caused by toxins to the host. Moreover, the vertebrate immune system also produces protease inhibitor molecules against the pathogens (Lai & Gallo, 2009). Pathogens, however, were shown to mimic host proteases (Doxey & McConkey, 2013) the primary function of which is to avoid the effect of protease inhibitors. This leads to the further increase of the adverse effects of the protease inhibitor tick toxins onto the vertebrate host.

Brown ear tick (*Rhipicephalus appendiculatus*) toxicosis is seemingly a strong evidence supporting the immunomodulation hypothesis of tick toxin molecules because after tick attachment, pathogens inside the host become very virulent and the reason behind that is not clarified (Njaa, 2017). However, it is not contradicting our antibiotic hypothesis because there is solid evidence that antibiotic treatment may in some specific cases increase the growth rate of different bacterial species directly (Capita et al., 2019). There are two possible explanations for this unexpected reaction of the antibiotic‐treated bacteria. First, with the higher reproduction rate they can increase their mutation rate to adapt and to survive (Ragheb et al., 2019). Second, with increasing the reproduction rate, they have higher chances for survival by the resulting higher number of individual bacterial cells. From the perspective of the host, on the other hand, the higher rate of division in bacterial pathogens is linked to increased virulence (Ewald, 1994).

This elevated virulence caused by bacterial reproduction triggered by the protease inhibitor tick toxin also contributes to the serious health consequences caused by the systematic detrimental effects of the protease inhibitor molecules onto the host protein metabolism. Hard ticks can only afford this harmful feeding side effect because they use only a single host in a given life stage and they do not depend on it after the relatively short time of their feeding compared to their life cycle.

## 3 TICK PARALYSIS

The two ticks most commonly associated with North American tick paralysis are the Rocky Mountain wood tick (*Dermacentor andersoni*) and the American dog tick (*Dermacentor variabilis*); however, 43 tick species have been implicated in human tick paralysis around the world (Gothe, Kunze, & Hoogstraal, 1979). In Australia, tick paralysis is caused by the eastern paralysis tick, *Ixodes holocyclus*, and the southern paralysis tick, *Ixodes cornuatus*, leading to many human cases but also affecting large number of dogs and cats. Prior to 1989, 20 fatal human cases were reported in Australia (Masina & Broady, 1999).

Paralysis toxins have a neurospecific effect, and they block neurotransmission (Grattan‐Smith, 1997) in the host leading to paralysis. Both some hard and some soft ticks can produce these toxins; in the latter case, only the larvae produce it that have the longest feeding time on the host. It is assumed that the evolutionary benefit of this trait is stopping the host's grooming activity and prevents the removal of the parasites. This hypothesis is also supported by empirical evidence. In experimentally induced tick paralysis, secretion of neurotoxin coincides with a definite repletion phase and in hard ticks is limited to females only (Mans et al., 2004). In all instances, paralysis coincides with the last rapid engorgement phase that is marked by the production and secretion of numerous protein products by the salivary glands and this is the state where the tick is the most susceptible to be removed by grooming. This indicates that ticks were selected for a trade‐off optimum and gained most of the possible benefits by stopping host grooming while reducing the adverse effects of host paralysis or death.

While paralysis is a generally observed host symptom, tick neurotoxins have diverse molecular characteristics. This indicates that the selective force to paralyse the host appeared many times independently (Mans et al., 2004) which is a sign of adaptive evolution. It was postulated earlier that paralysis induced by soft ticks is distinct from paralysis induced by hard ticks because soft tick paralysis is only caused by larvae (Mans et al., 2004). However, it seems to be a direct consequence of the argasid life cycle, because the typical adult and nymphal soft ticks take blood meal for only a very short period of time (seconds to minutes), while their larval stages have a longer (several days) attachment similarly to all hard tick stages (Sonenshine & Roe, 2013).

## 4 SAND TAMPAN TOXICOSIS

There is a very enigmatic third type of toxicosis caused by the soft tick sand tampan (*Ornithodoros savignyi*; Mans et al., 2002). Sand tampans by their concerted attack in large numbers are able to paralyse and kill sizeable mammals, especially penned livestock, by introducing toxins during feeding, mainly through coxal gland secretions, leading to symptoms similar to those of anaphylactic shock in older animals (Horak et al., 2015). The toxin directly attacks the heart of the host organism in mouse models causing tachycardia and other forms of heart complications. It is suggested to result in great loss for similar reasons especially in the population of bovines (Mans et al., 2002). This kind of toxicosis seems very paradoxical and does not fit into any of the previously mentioned examples. It is important to note that sand tampans take their blood meal during the night when the host organisms sleep. At the state of sleep, heart rate falls to a very low level (Åkerstedt & Nilsson, 2003) which is disadvantageous for the parasite because of the decreasing amount of blood. Therefore, producing a molecule to elevate the beating of the heart is clearly an adaptive strategy, and by this, the existence of the sand tampan toxicosis can also be explained from an evolutionary standpoint.

This type of toxicosis also differs from hard tick‐induced toxicosis in the amount of toxin used. Hard ticks take blood meal for several days and inject their toxic molecules in large quantities into their hosts. In contrast, soft ticks take short blood meals as adults; therefore, they can inject a relatively small amount of their toxins. This is probably the reason behind the difference between the more specific cardiac effect of sand tampan toxicosis compared to the systemic antimicrobial effect of some hard tick toxicoses.

## 5 TICK BITE NECROSIS

In several tick species, a skin manifestation after tick bite has been reported called tick bite necrosis (Kallini & Khachemoune, 2017). This is an enigmatic host reaction similar to the one occurring in some snake and spider bites and during some bacterial infections (Heise, Ruha, Padilla‐Jones, Hayek, & Gerkin, 2018; Hobbs & Harrell, 1989; Puvanendran, Huey, & Pasupathy, 2009). But the background of the development of these skin manifestations is essentially different in ticks and predators. Venomous predators induce necrotic lesions with their bites by actively producing necrotoxins that trigger necroptotic reaction in order to immobilize or kill their prey. Contrary to this, in the case of ticks we assume that the necrotic lesions develop without the injection of toxic molecules into the host organism. The question arises: What is the adaptive function of these lesions in the parasitic ticks? The prophylaxis theory of allergy developed by Margie Profet might help us understand. It states that allergic reaction is an immediate adaptive response (Palm, Rosenstein, & Medzhitov, 2012; Profet, 1991). Necrosis could have a similar protective host function, and the recently discovered mechanism called necroptosis supports this suggestion. Necroptosis is an alternative of apoptosis, the well‐known programmed cell death process. It is a radical host mechanism to protect from the invasion of intracellular pathogens (Linkermann & Green, 2014; Wu, Liu, & Li, 2012). Thus, we hypothesize that necrotic tissue lesion produced by the host against soft and hard tick bites is not triggered by a toxin as in venomous predators but by the plenty of pathogens they transmit.

Interestingly, these skin lesions occur typically after the bites of soft ticks but are rare in the case of hard ticks. The possible explanation to this difference could be that the duration of blood feeding is maximally one hour in the case of soft ticks (Ribeiro & Valenzuela, 2011), while hard ticks attach to the host for several days; therefore, it is essential for them to suppress the necroptotic reaction in their case which process is an immediate protective reaction to stop the invasion of pathogenic agents (Linkermann & Green, 2014). Further research would be needed to find these hypothetical molecules in hard ticks that may be able to neutralize host necroptotic reactions.

## 6 POTENTIAL MEDICAL APPLICATIONS OF TICK TOXINS

As we theorize based on many published studies, the main function of tick ixovotoxins with protease inhibitor activity is most probably to systemically attack the tick‐transmitted pathogens inside the vertebrate host. Thus, this tick toxin can be considered as an ancient, several hundred millions of years old (Sonenshine & Roe, 2013) natural antibiotics. In the last decades, antibiotic resistance has been one of the most challenging problems of the 21st century and to find new types of drugs in the arms race with our pathogens has a priority in medical profession. With the advance of molecular pharmacology, it is possible that these tick toxins can serve as excellent new candidates for future effective antibiotics especially if we can control the adverse secondary effects (Figure 1).

Sand tampan toxins might be effective heart medicines in the future, and the adverse effects of these molecules in the host organisms in the form of tachycardia could be turned into life‐saving drugs. The dangerous paralysis toxins injected into the host could be an effective solution to treat tremors which is a typical symptom in diseases like Parkinson's disease (Hallett, 2012). The defensins employed by *Ornithodoros savignyi* are being studied for developing multifunctional peptides. Shorter peptides derived from the defensin isoform 2 (OsDef2) have useful antibacterial, antioxidant and cytotoxic properties (Prinsloo et al., 2013). At the end of the list of toxicoses, it is important to mention the frightening medical condition of necrosis. This state is a frequent complication after some bacterial infections, snake, spider and soft tick bite, and can be life‐threatening. As we previously discussed in this article, necrosis after tick bite is much more common in soft than hard ticks and we argued that the reason for this is that soft ticks take blood meal for a very short period of time, while hard ticks attach to their host for several days, up to two weeks. An early onset of necrotic tissue damage can be fatal for hard ticks; therefore, any mechanism that can inhibit the necroptotic process should be strongly favoured by natural selection. If these theoretical suppressor molecules really exist, they can be directly turned against the medical condition of extensive necrosis in a form of a newly developed effective drug.

We tend to look at ticks as one of our primary enemies and eradicating them is the main goal for many. Ticks, however, thank to their perfect adaptation to parasitic lifestyle that they were selected for during their long evolutionary history, possess biological tools that scientists have not yet developed. Making use of their evolutionary applications might turn our foes to friends that would help us conquer some of our medical challenges.

## CONFLICT OF INTEREST

None declared.

## References

[eva13123-bib-0001] Åkerstedt, T. , & Nilsson, P. M. (2003). Sleep as restitution: An introduction. Journal of Internal Medicine, 254(1), 6–12. 10.1046/j.1365-2796.2003.01195.x 12823638

[eva13123-bib-0002] Blisnick, A. A. , Foulon, T. , & Bonnet, S. I. (2017). Serine protease inhibitors in ticks: an overview of their role in tick biology and tick‐borne pathogen transmission. Frontiers in Cellular and Infection Microbiology, 7, 199 10.3389/fcimb.2017.00199 28589099 PMC5438962

[eva13123-bib-0003] Bowman, A. S. , & Nuttall, P. A. (2004). Ticks: Biology, Disease and Control. Cambridge, UK: Cambridge University Press.

[eva13123-bib-0004] Burgdorfer, W. , Hayes, S. F. , & Corwin, D. (1989). Pathophysiology of the lyme disease spirochete, borrelia burgdorferi, in ixodid ticks. Reviews of Infectious Diseases, 11(Supplement_6), S1442–S1450. 10.1093/clinids/11.Supplement_6.S1442 2682956

[eva13123-bib-0005] Buysse, M. , Plantard, O. , McCoy, K. D. , Duron, O. , & Menard, C. (2019). Tissue localization of Coxiella‐like endosymbionts in three European tick species through fluorescence in situ hybridization. Ticks and Tick‐Borne Diseases, 10(4), 798–804. 10.1016/j.ttbdis.2019.03.014 30922601

[eva13123-bib-0006] Cabezas‐Cruz, A. , & Valdés, J. J. (2014). Are ticks venomous animals? Frontiers in Zoology, 11, 47 10.1186/1742-9994-11-47 25006341 PMC4085379

[eva13123-bib-0007] Capita, R. , Vicente‐Velasco, M. , Rodríguez‐Melcón, C. , García‐Fernández, C. , Carballo, J. , & Alonso‐Calleja, C. (2019). Effect of low doses of biocides on the antimicrobial resistance and the biofilms of Cronobacter sakazakii and Yersinia enterocolitica. Scientific Reports, 9(1), 15905 10.1038/s41598-019-51907-1 31685860 PMC6828698

[eva13123-bib-0008] Cordeiro, F. A. , Amorim, F. G. , Anjolette, F. A. P. , & Arantes, E. C. (2015). Arachnids of medical importance in Brazil: Main active compounds present in scorpion and spider venoms and tick saliva. Journal of Venomous Animals and Toxins including Tropical Diseases, 21(1), 24 10.1186/s40409-015-0028-5 PMC4535291 26273285

[eva13123-bib-0009] Corral‐Rodríguez, M. A. , Macedo‐Ribeiro, S. , Barbosa Pereira, P. J. , & Fuentes‐Prior, P. (2009). Tick‐derived Kunitz‐type inhibitors as antihemostatic factors. Insect Biochemistry and Molecular Biology, 39(9), 579–595. 10.1016/j.ibmb.2009.07.003 19631744

[eva13123-bib-0010] Dai, S.‐X. , Zhang, A.‐D. , & Huang, J.‐F. (2012). Evolution, expansion and expression of the Kunitz/BPTI gene family associated with long‐term blood feeding in Ixodes Scapularis. BMC Evolutionary Biology, 12(1), 4 10.1186/1471-2148-12-4 22244187 PMC3273431

[eva13123-bib-0011] Doxey, A. C. , & McConkey, B. J. (2013). Prediction of molecular mimicry candidates in human pathogenic bacteria. Virulence, 4(6), 453–466. 10.4161/viru.25180 23715053 PMC5359739

[eva13123-bib-0012] Ewald, P. W. (1994). Evolution of infectious disease, New York, NY: Oxford University Press.

[eva13123-bib-0013] Gothe, R. , Kunze, K. , & Hoogstraal, H. (1979). Review article: the mechanisms of pathogenicity in the tick paralyses. Journal of Medical Entomology, 16(5), 357–369. 10.1093/jmedent/16.5.357 232161

[eva13123-bib-0014] Grattan‐Smith, P. (1997). Clinical and neurophysiological features of tick paralysis. Brain, 120(11), 1975–1987. 10.1093/brain/120.11.1975 9397015

[eva13123-bib-0015] Hajdusek, O. , Sima, R. , Ayllon, N. , Jalovecka, M. , Perner, J. , De La Fuente, J. , & Kopacek, P. (2013). Interaction of the tick immune system with transmitted pathogens. Frontiers in Cellular and Infection Microbiology, 3, 10.3389/fcimb.2013.00026 PMC3712896 23875177

[eva13123-bib-0016] Hallett, M. (2012). Parkinson’s disease tremor: Pathophysiology. Parkinsonism & Related Disorders, 18, S85–S86. 10.1016/S1353-8020(11)70027-X 22166464

[eva13123-bib-0017] Harris, E. K. , Verhoeve, V. I. , Banajee, K. H. , Macaluso, J. A. , Azad, A. F. , & Macaluso, K. R. (2017). Comparative vertical transmission of Rickettsia by Dermacentor variabilis and Amblyomma maculatum. Ticks and Tick‐Borne Diseases, 8(4), 598–604. 10.1016/j.ttbdis.2017.04.003 28433729 PMC5702269

[eva13123-bib-0018] Heise, C. W. , Ruha, A.‐M. , Padilla‐Jones, A. , Hayek, C. T. , & Gerkin, R. D. (2018). Clinical predictors of tissue necrosis following rattlesnake envenomation. Clinical Toxicology, 56(4), 281–284. 10.1080/15563650.2017.1371311 28885114

[eva13123-bib-0019] Hobbs, G. D. , & Harrell, R. E. (1989). Brown recluse spider bites: A common cause of necrotic arachnidism. The American Journal of Emergency Medicine, 7(3), 309–312. 10.1016/0735-6757(89)90178-2 2712898

[eva13123-bib-0020] Horak, I. G. , Jordaan, A. J. , Nel, P. J. , van Heerden, J. , Heyne, H. , & van Dalen, E. M. (2015). Distribution of endemic and introduced tick species in Free State Province, South Africa. Journal of the South African Veterinary Association, 86(1), 9 10.4102/jsava.v86i1.1255 PMC6138067 26244582

[eva13123-bib-0021] Kallini, J. R. , & Khachemoune, A. (2017). Ticks and tick bites presenting as “Funny Moles”: a review of different presentations and a focus on tick‐borne diseases. The Journal of Clinical and Aesthetic Dermatology, 10(3), 46–50. 28360969 PMC5367882

[eva13123-bib-0022] Lai, Y. , & Gallo, R. L. (2009). AMPed Up immunity: How antimicrobial peptides have multiple roles in immune defense. Trends in Immunology, 30(3), 131–141. 10.1016/j.it.2008.12.003 19217824 PMC2765035

[eva13123-bib-0023] Linkermann, A. , & Green, D. R. (2014). Necroptosis. The New England Journal of Medicine, 370(5), 455–465. 10.1056/NEJMra1310050 24476434 PMC4035222

[eva13123-bib-0024] Mans, B. J. , Gothe, R. , & Neitz, A. W. H. (2004). Biochemical perspectives on paralysis and other forms of toxicoses caused by ticks. Parasitology, 129(S1), S95–S111. 10.1017/S0031182003004670 15938507

[eva13123-bib-0025] Mans, B. J. , Steinmann, C. M. L. , Venter, J. D. , Louw, A. I. , & Neitz, A. W. H. (2002). Pathogenic mechanisms of sand tampan toxicoses induced by the tick, Ornithodoros savignyi. Toxicon, 40(7), 1007–1016. 10.1016/S0041-0101(02)00098-3 12076655

[eva13123-bib-0026] Masina, S. , & Broady, K. W. (1999). Tick paralysis: Development of a vaccine. International Journal for Parasitology, 29(4), 535–541. 10.1016/S0020-7519(99)00006-5 10428629

[eva13123-bib-0027] Mukherjee, A. K. , Mackessy, S. P. , & Dutta, S. (2014). Characterization of a Kunitz‐type protease inhibitor peptide (Rusvikunin) purified from Daboia russelii russelii venom. International Journal of Biological Macromolecules, 67, 154–162. 10.1016/j.ijbiomac.2014.02.058 24632346

[eva13123-bib-0028] Niebylski, M. L. , Peacock, M. G. , & Schwan, T. G. (1999). Lethal effect of rickettsia rickettsii on its tick vector (Dermacentor andersoni). Applied and Environmental Microbiology, 65(2), 773–778. 10.1128/AEM.65.2.773-778.1999 9925615 PMC91094

[eva13123-bib-0029] Njaa, B. L. (2017). Chapter 20—The Ear In J. F. Zachary (Ed.), Pathologic basis of veterinary disease, 6th edn (pp. 1223–1264.e1). St Louis, MO: Elsevier 10.1016/B978-0-323-35775-3.00020-5

[eva13123-bib-0030] Palm, N. W. , Rosenstein, R. K. , & Medzhitov, R. (2012). Allergic host defenses. Nature, 484(7395), 465–472. 10.1038/nature11047 PMC3596087 22538607

[eva13123-bib-0031] Prinsloo, L. , Naidoo, A. , Serem, J. , Taute, H. , Sayed, Y. , Bester, M. , … Gaspar, A. (2013). Structural and functional characterization of peptides derived from the carboxy‐terminal region of a defensin from the tick *Ornithodoros savignyi*: Peptides derived from the *c* ‐terminal of a tick defensin. Journal of Peptide Science, 19(5), 325–332. 10.1002/psc.2505 23553969

[eva13123-bib-0032] Profet, M. (1991). The function of allergy: Immunological defense against toxins. The Quarterly Review of Biology, 66(1), 23–62. 10.1086/417049 2052671

[eva13123-bib-0033] Puvanendran, R. , Huey, J. C. M. , & Pasupathy, S. (2009). Necrotizing fasciitis. Canadian Family Physician, 55(10), 981–987. 19826154 PMC2762295

[eva13123-bib-0034] Rachinsky, A. , Guerrero, F. D. , & Scoles, G. A. (2007). Differential protein expression in ovaries of uninfected and Babesia‐infected southern cattle ticks, Rhipicephalus (Boophilus) microplus. Insect Biochemistry and Molecular Biology, 37(12), 1291–1308. 10.1016/j.ibmb.2007.08.001 17967348

[eva13123-bib-0035] Ragheb, M. N. , Thomason, M. K. , Hsu, C. , Nugent, P. , Gage, J. , Samadpour, A. N. , … Merrikh, H. (2019). Inhibiting the evolution of antibiotic resistance. Molecular Cell, 73(1), 157–165.e5. 10.1016/j.molcel.2018.10.015 30449724 PMC6320318

[eva13123-bib-0036] Ribeiro, J. M. C. , & Valenzuela, J. G. (2011). CHAPTER 8—Vector Biology In R. L. Guerrant , D. H. Walker , & P. F. Weller (Eds.), Tropical infectious diseases: principles, pathogens and practice, 3^rd^ ed, (pp. 45 –51). 10.1016/B978-0-7020-3935-5.00008-2

[eva13123-bib-0037] Socolovschi, C. , Mediannikov, O. , Raoult, D. , & Parola, P. (2009). The relationship between spotted fever group Rickettsiae and Ixodid ticks. Veterinary Research, 40(2), 34 10.1051/vetres/2009017 19358804 PMC2695030

[eva13123-bib-0038] Sonenshine, D. E. , & Roe, R. M. (2013). Biology of Ticks Volume 2. New York, NY: Oxford University Press.

[eva13123-bib-0039] Wu, W. , Liu, P. , & Li, J. (2012). Necroptosis: An emerging form of programmed cell death. Critical Reviews in Oncology/Hematology, 82(3), 249–258. 10.1016/j.critrevonc.2011.08.004 21962882

